# Intraepithelial neutrophils in pediatric severe asthma are associated with better lung function

**DOI:** 10.1016/j.jaci.2016.09.022

**Published:** 2017-06

**Authors:** Cecilia K. Andersson, Alexandra Adams, Prasad Nagakumar, Cara Bossley, Atul Gupta, Daphne De Vries, Afiqah Adnan, Andrew Bush, Sejal Saglani, Clare M. Lloyd

**Affiliations:** a Inflammation, Repair and Development Section, National Heart and Lung Institute, Imperial College, London, United Kingdom; b Respiratory Paediatrics, the Royal Brompton and Harefield NHS Trust, Sydney Street, London, United Kingdom

**Keywords:** Pediatric asthma, severe therapy-resistant asthma, IL-17A, IL-17A receptor, neutrophils, ACT, Asthma control test, BAL, Bronchoalveolar lavage, PBEC, Primary bronchial epithelial cell, STRA, Severe therapy-resistant asthma

## Abstract

**Background:**

Neutrophils and IL-17A have been linked mechanistically in models of allergic airways disease and have been associated with asthma severity. However, their role in pediatric asthma is unknown.

**Objectives:**

We sought to investigate the role of neutrophils and the IL-17A pathway in mediating pediatric severe therapy-resistant asthma (STRA).

**Methods:**

Children with STRA (n = 51; age, 12.6 years; range, 6-16.3 years) and controls without asthma (n = 15; age, 4.75 years; range, 1.6-16 years) underwent clinically indicated fiberoptic bronchoscopy, bronchoalveolar lavage (BAL), endobronchial brushings, and biopsy. Neutrophils, IL-17A, and IL-17RA–expressing cells and levels of IL-17A and IL-22 were quantified in BAL and biopsies and related to clinical features. Primary bronchial epithelial cells were stimulated with IL-17A and/or IL-22, with and without budesonide.

**Results:**

Children with STRA had increased intraepithelial neutrophils, which positively correlated with FEV_1_ %predicted (*r* = 0.43; *P* = .008). Neutrophil^high^ patients also had better symptom control, despite lower dose maintenance inhaled steroids. Submucosal neutrophils were not increased in children with STRA. Submucosal and epithelial IL-17A–positive cells and BAL IL-17A and IL-22 levels were similar in children with STRA and controls. However, there were significantly more IL-17RA–positive cells in the submucosa and epithelium in children with STRA compared with controls (*P* = .001). Stimulation of primary bronchial epithelial cells with IL-17A enhanced mRNA expression of IL-17RA and increased release of IL-8, even in the presence of budesonide.

**Conclusions:**

A proportion of children with STRA exhibit increased intraepithelial airway neutrophilia that correlated with better lung function. STRA was also characterized by increased airway IL-17RA expression. These data suggest a potential beneficial rather than adverse role for neutrophils in pediatric severe asthma pathophysiology.

Severe asthma is heterogeneous and can be divided into subphenotypes.^1^, ^2^, ^3^ Severe therapy-resistant asthma (STRA) affects a small proportion of children with asthma and is characterized by persistent symptoms, acute severe exacerbations, and/or fixed airflow obstruction despite treatment with high-dose steroid therapy, and after modifiable factors such as poor adherence and persistent allergen exposure have been addressed.^4^ Because T_H_2-mediated eosinophilic asthma generally responds well to steroid treatment, the lack of responsiveness to steroids in STRA suggests an alternative inflammatory pathway in these children. We have previously shown that children with STRA exhibit airway eosinophilia and remodeling, but a paucity of classical T_H_2 cytokines (IL-4, IL-5, and IL-13).^5^

In adults, airway neutrophils have been associated with increased asthma severity^1^, ^6^, ^7^ but their functional role in mediating disease pathophysiology is unclear. Elevated levels of IL-17A have been reported in sputum, bronchoalveolar lavage (BAL) fluid, and peripheral blood^8^, ^9^, ^10^, ^11^ in those with severe asthma and have been implicated in pathogenesis.^10^, ^12^ In contrast, little is known about the role of neutrophils and the IL-17 pathway in children with STRA. IL-17A can induce bronchial epithelial secretion of proinflammatory cytokines including the neutrophil chemoattractants GRO and IL-8. It is proposed that IL-17A can therefore induce neutrophilic airway inflammation and promote steroid resistance in adults with severe asthma.^13^ Moreover, IL-17A may also be involved in airway remodeling. IL-17A has been reported to increase airway smooth muscle contraction on methacholine stimulation and drive migration of airway smooth muscle cells in murine models of allergic inflammation and in patients with asthma.^9^, ^14^, ^15^ IL-17A binds to the receptor subunits IL-17RA and C, which are expressed on epithelial and mesenchymal cells as well as on some immune cell populations such as lymphocytes, dendritic cells, and monocytes/macrophages in the lung.^16^ Mice that lack IL-17RA produce less CXCL1 and CXCL2 on pulmonary challenge with *Klebsiella pneumoniae*^17^ and IL-17RA signaling is also necessary for host defense against *Candida albicans*.^18^ In 2 mouse models, blocking IL-17R and IL-17RB reduced airway inflammation and airway hyperreactivity.^14^ T_H_17 cells also secrete IL-22, and IL-22 mRNA is elevated in PBMCs in pediatric patients with severe asthma, and rhinitis.^19^ Although IL-22 has been described as a proinflammatory cytokine causing airway hyperreactivity in mice and remodeling in both epithelial and airway smooth muscle cells in humans, it has also been shown to reduce inflammation by suppressing cytokine production from epithelial cells. Taken together, data suggest that IL-22 plays different roles in various phases of airway inflammation.^20^, ^21^

We have previously reported that there was no increase in mucosal or BAL neutrophils in patients with STRA. However, when reexamining biopsy slides, we noted that some but not all of these children had intraepithelial neutrophils. We therefore hypothesized that intraepithelial neutrophils, together with elevated IL-17A and IL-22, would be associated with worse asthma severity. We determined the extent of neutrophilic inflammation in a new cohort of children with STRA and investigated IL-17A and IL-22 levels and their cellular sources in BAL. We assessed the response of bronchial epithelial cells to IL-17A, IL-22, and steroids in patients with STRA compared with controls. The findings were then related to key clinical features and airway remodeling and were confirmed in archived biopsies from an older cohort of children with STRA to look specifically at intraepithelial neutrophils, which have not previously been reported.

## Methods

### Subjects

Fifty-one school-aged children with STRA^22^ (age, 12.6 years; range, 6-16.3 years) were recruited from the Royal Brompton Hospital. They underwent detailed clinical assessments including spirometry, exhaled nitric oxide measurements, and symptom scores followed by a clinically indicated bronchoscopy, endobronchial brushings, BAL, and endobronchial biopsies to characterize airway pathology and develop customized treatment plans.^4^ All children had previously undergone a detailed assessment to ensure any modifiable factors such as poor adherence or persistent allergen exposure had been addressed before the bronchoscopy.^23^ *Atopy* was defined as at least 1 positive specific IgE radioallergosorbent test result (≥0.35 kUI/L) to aeroallergens (house dust mite, cat, dog, and grass pollen) and quantified as the sum of specific IgE levels of these aeroallergens. Fifteen control subjects without asthma (age, 4.75 years; range, 1.6-16 years) were either (A) having a bronchoscopy to investigate upper airway symptoms and agreed to extra research samples being taken or (B) undergoing general anesthesia for cardiac catheterization and agreed to have a research bronchoscopy at the same time (for details, see Table I and Table E2 in this article's Online Repository at www.jacionline.org). The study was approved by the National Research Ethics Service Committee London - Chelsea, and informed parental consent and child assent were obtained. In addition to the above-mentioned cohort, findings relating to intraepithelial inflammation were confirmed in biopsies obtained from a previously published cohort^5^ of children with STRA (n = 21) and controls (n = 5). In all figures, the latter will appear as gray symbols and their clinical details are described in detail in this article's Online Repository and in Table E1 in the Online Repository at www.jacionline.org. Inclusion of both cohorts of children is of vital importance to the strength of the findings because we were able to replicate the finding even in this second cohort. There is no duplication of the data because we merely used the archived biopsy samples to examine neutrophils, which had not previously been examined in these samples. Further details of investigations are given in the Methods section in this article's Online Repository at www.jacionline.org.

**Table I:** Demographic characteristics of children with STRA and control patients undergoing bronchoscopy

Characteristic	STRA (n = 51)	Controls without asthma∗ (n = 15)	*P* value
Atopy	45 of 51 (88.2%)	1 of 15 (6.6%)	.005
Male:female	30:21	8:7	
Age (y)	12.6 (6 to 16.3)	4.75 (1.6 to 16)	.005
Duration of symptoms (y)	7 (3.5 to 14.3)		
Weight (kg)	41.7 (22.3 to 99.9)	19.2 (8.9 to 68)	
Weight *z* score	0.5 (−3.4 to 3.7)	0.4 (−3.1 to 3.4)	
Height (cm)	150 (106 to 188)		
Height *z* score	0.02 (−3.9 to 2.88)		
Intubation for asthma	4 of 51 (7.8%)		
Total IgE (IU/mL)	419 (20 to 4867)	46.5 (1 to 210)	.003
Sum of inhalant specific IgE (IU/mL)	7.4 (0 to 321)	0.54 (0 to 20)	.1
Sum of all specific IgE (IU/mL)	11.3 (0 to 321)	1.1 (0 to 20)	.2
BAL neutrophils (%)†	3.7 (1 to 21.7)	5.4 (4 to 9)	.35
BAL eosinophils (%)‡	3 (0.3 to 23)	2 (0 to 5)	.17
Blood eosinophils (%)	7.5 (0 to 21.4)	3.5 (0.9 to 9.8)	.06
Blood neutrophils (%)	51 (6 to 87)	49 (31 to 71)	.4
ACT score	13 (5 to 23)		
ACT normal (>19/25)	10 of 51 (19.6%)		
Baseline FEV_1_ (L)	1.75 (0.45 to 3.95)		
Baseline % predicted FEV_1_§	89.4 (24 to 126)		
Number FEV_1_ “normal” (>80% predicted)	31 of 51 (60.7%)		
Baseline FVC (L)	2.36 (1.37 to 5.39)		
Baseline % predicted FVC	99.5 (63 to 133)		
Baseline bronchodilator reversibility (%)	11.4 (−3 to 66.7)		
Baseline Feno_50_ (ppb)‖	46.2 (5.4 to 164.8)		
Medications			
Daily dose inhaled¶ corticosteroid (μg/d) budesonide equivalent	1400 (500 to 2000)	0 (0 to 200)	
Leukotriene receptor antagonist	46 of 51 (90.1%)	0	
Systemic corticosteroids	6 of 51 (11.7%)	0	
Daily dose (mg/d)	6.25 (2.5 to 20)		
Theophylline	4 of 51 (7.8%)	0	

Data presented as median (range).

∗ Eighty percent of the controls without asthma had symptoms such as stridor (tracheal stenosis, laryngotracheomalacia), reflux, dry cough, or hemoptysis. The remaining 20% were undergoing general anesthesia for elective cardiac catheterization and had agreed to a research bronchoscopy. For details of diagnosis in controls without asthma, see Table E2. Differences between groups were assessed by Mann-Whitney test where *P* < .05 is significant.

† BAL neutrophils (<3% is normal).

‡ BAL eosinophils (<3% is normal).

§ FEV_1_: presented as percentage predicted.

|| Feno: fractional exhaled nitric oxide measured at 50 L/min.

¶ ICS: inhaled corticosteroids/d.

### Epithelial culture and stimulation

Primary bronchial epithelial cells (PBECs) were seeded into tissue culture flasks containing bronchial epithelial growth medium and used at passage 3 for all experiments. PBECs were stimulated with recombinant human IL-17A (eBioscience, San Diego, Calif) and IL-22 (eBioscience) alone, or to cells pretreated with budesonide (Breath Limited, Barnstaple, United Kingdom) as stated in Zijlstra et al.^24^ Culture supernatants were collected after 24 hours and samples for mRNA extraction were harvested after 8-hour stimulation (see this article's Online Repository).

### Quantification of cytokines

Cell culture supernatant was collected and cytokines analyzed using ELISA and Milliplex human cytokine panel I and II (see this article's Online Repository).

### Cytomix

BAL cytokine quantification was performed using Flowcytomix Human T_H_1/T_H_2/T_H_9/T_H_17/T_H_22 13 plex multiplex (eBioscience) (see this article's Online Repository).

### Flow cytometry

Lymphocytes used for compensations and fluorescence minus ones were extracted from peripheral blood and cells from the airway lumen were obtained after centrifuge of BAL fluid. Cells were stained for extracellular markers CD3, CD4, CD8, CD161, and γδTCR and intracellular IL-17A (eBiosciences, mouse anti-human 5 μL per well) (see this article's Online Repository).

### RNA extraction and real-time PCR

Total RNA was extracted from epithelial cells using the Qiagen RNeasy Mini Kit (Qiagen, Hilden, Germany). cDNA was synthesized from 500 ng of total RNA and analyzed by using High Capacity cDNA Reverse Transcription Kit (Applied Biosystems, Foster City, Calif) (see this article's Online Repository).

### Histopathology

Endobronchial biopsies were processed to paraffin. Five-micrometer sections were stained with hematoxylin and eosin and used to assess morphology and consecutive sections were used for Masson's trichrome (Sigma Aldrich, Gillingham, United Kingdom) for collagen staining, Congo red for staining of eosinophils, and immunohistochemical staining for IL-17A, IL-17RA, and neutrophil elastase (see this article's Online Repository).

### Statistical analysis

Sample size was opportunistic because there are no data to inform a power calculation. Nonparametric tests including Mann-Whitney *U* test and Kruskal Wallis test with Bonferroni *post hoc* test were used to detect differences between 2 groups or more than 2 groups, respectively, using GraphPad Prism 6 (GraphPad Software, La Jolla, Calif). Correlations were assessed using the Spearman rank correlation test. *P* value of less than .05 was considered significant.

## Results

### Patients' demographic characteristics

Clinical characteristics are presented in Table I. BAL bacterial culture was positive in 9 of 51 (15.6%) and BAL viral PCR was positive in 5 of 51 (9.8%) patients with STRA. There was a parental report of exposure to tobacco smoke in 15 of 41 (37%) patients with STRA, which was confirmed using urinary cotinine levels.

### A subgroup of STRA is characterized by increased intraepithelial neutrophils

**Fig 1:**
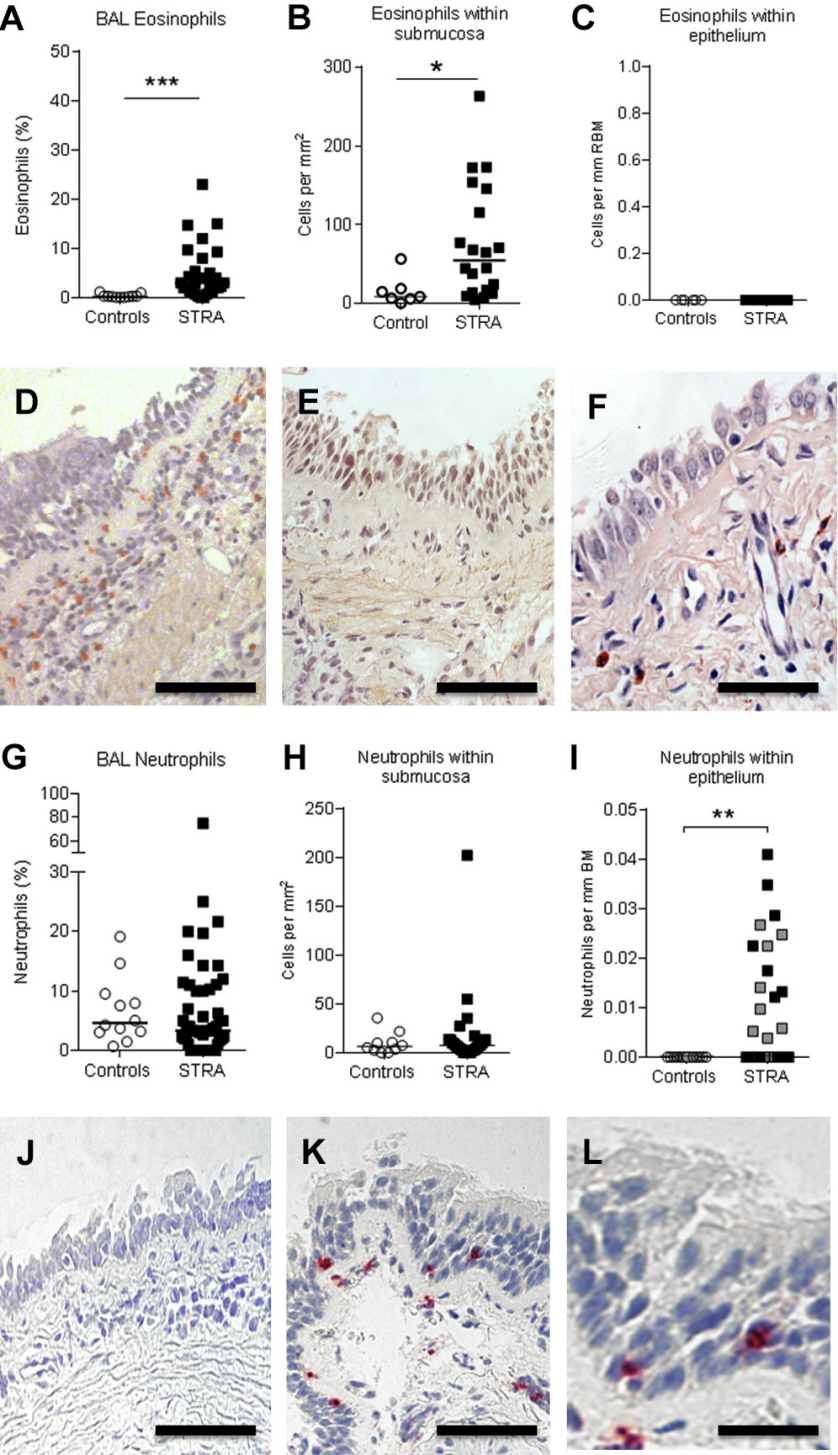
Eosinophils and neutrophils in different lung compartments. Eosinophil percentage in BAL **(A)** (controls n = 11, STRA n = 31), numbers per area of submucosa **(B)** (controls without asthma n = 7, STRA n = 20), and intraepithelial eosinophils per length of reticular basement membrane **(C)** (controls n = 7, STRA n = 20). Representative micrographs of Congo Red stain for eosinophils in controls without asthma **(D)** and patients with STRA **(E)**. **F,** High magnification picture of epithelium with no eosinophils in patients with STRA. Neutrophil percentage in BAL **(G)** (controls without asthma n = 12, STRA n = 52), numbers per area of submucosa **(H)** (controls without asthma n = 10, STRA n = 28), and proportion of intraepithelial neutrophils **(I)** (controls without asthma n = 13, STRA n = 37). Representative micrographs of immunohistochemical stain for elastase in controls without asthma **(J)** and patients with STRA **(K)**. **L,** High magnification picture of intraepithelial neutrophils in patients with STRA. Scale bar: 200 μm (Fig 1, *D* and *J*), 100 μm (Fig 1, *E* and *K*), 20 μm (Fig 1, *F*), and 10 μm (Fig 1, *L*). Statistical significance between controls without asthma and patients with STRA was tested using Mann-Whitney test. *P < .05, **P < .01, and ***P < .001.

Previous findings from our group^5^ that showed increased BAL and submucosal eosinophils in children with STRA compared with controls were confirmed in the present study (Fig 1, *A* and *B* and *D* and *E*). However, when investigating intraepithelial eosinophils in both the present and the previous cohort,^5^ we found no eosinophils in the epithelium of controls or children with STRA (Fig 1, *C* and *F*). Because severe asthma in adult patients^13^ and in murine models^10^, ^25^, ^26^, ^27^ has been associated with neutrophilia, we quantified the number of neutrophils in the submucosa and epithelium in endobronchial biopsies from children with STRA and controls. In keeping with our previous findings,^5^ neutrophil counts were not increased in the submucosa of children with STRA compared with controls (*P* = .9; Fig 1, *G* and *H* and *J* and *K*). However, intraepithelial neutrophils, expressed both as proportion of all neutrophils in the biopsy and as neutrophils per length basement membrane, were significantly higher in children with STRA than in controls (*P* = .01 and .007, respectively) (Fig 1, *I* and *L*). We also confirmed the increase in intraepithelial neutrophils in STRA in biopsies obtained from a previous cohort^5^ of children with STRA (Fig 1, *I*, shown as gray symbols; for clinical details of these patients, see Table E1). The patients with intraepithelial neutrophils (referred to as Neutrophil^high^) had a median of 0.02 neutrophils per length of reticular basement membrane (range, 0.004-0.04) compared with Neutrophil^low^ patients (0; range, 0-0) (*P* = .007). There was no difference in the number of intraepithelial neutrophils between patients with STRA treated with maintenance oral and inhaled steroids compared with those treated with only inhaled steroids (*P* = .8).

### Children with STRA did not have increased BAL or tissue IL-17A

**Fig 2:**
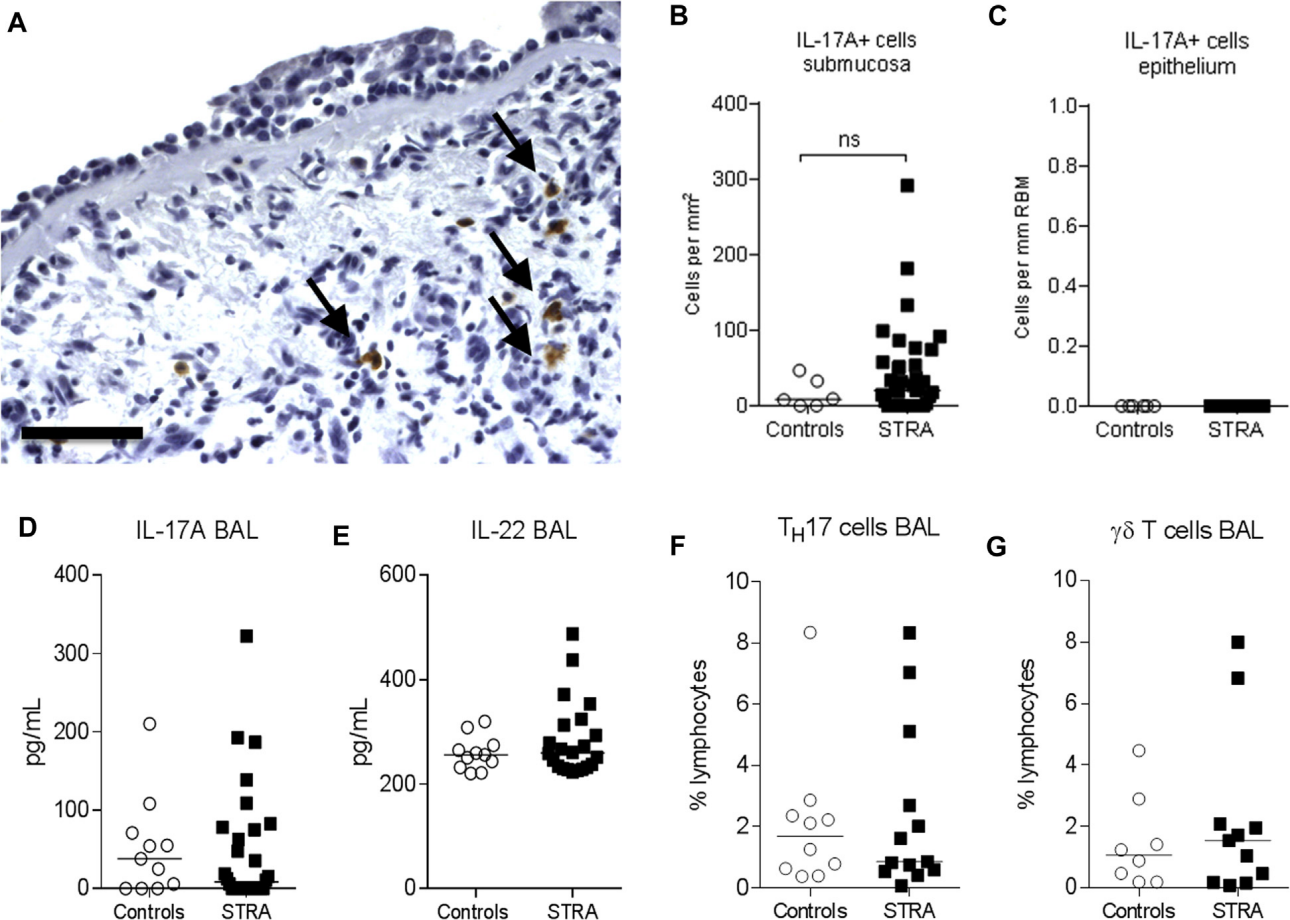
Sources and expression of IL-17A in children with STRA compared with controls without asthma. Representative micrograph of immunohistochemical stain for IL-17A in children with STRA **(A)**. Scale bar: 100 μm. Quantification of the number of IL-17A–expressing cells per area in submucosa **(B)** (controls without asthma n = 6, STRA n = 39) and bronchial epithelium **(C)** (controls without asthma n = 6, STRA n = 32). BAL levels of IL-17A **(D)** (controls without asthma n = 11, STRA n = 23) and IL-22 **(E)** (controls without asthma n = 11, STRA n = 23) in controls without asthma and children with STRA. Percentages of IL-17A–expressing T_H_17 **(F)** (controls without asthma n = 10, STRA n = 13) and gamma delta **(G)** (controls without asthma n = 8, STRA n = 11) lymphocytes in children with STRA compared with controls without asthma. Statistical significance between controls without asthma and children with STRA was tested using Mann-Whitney test. *NS*, Not significant.

To investigate any relationship between neutrophils and airway IL-17A in children with STRA, endobronchial biopsies were stained for IL-17A. IL-17A expression was observed in small mononuclear cells as well as in a larger mononuclear cell population in the adventitia of the bronchial wall, but there was no difference in the number of IL-17A–positive cells between controls and patients with STRA (Fig 2, *A* and *B*). No IL-17A expression was seen within the epithelium (Fig 2, *A* and *C*). Although undetectable in most samples, IL-17A levels in BAL were similar in children with STRA and controls (Fig 2, *D*). IL-22 was detected in BAL fluid in 15 of 25 controls (60%) and 25 of 37 patients with STRA (68%). However, IL-22 levels were similar in both groups (Fig 2, *E*). There was also no difference in the proportion of T_H_17 (CD4^+^IL-17^+^) (Fig 2, *F*) or IL-17^+^ γδT cells (Fig 2, *G*) in BAL from children with STRA compared with controls.

### Children with STRA had increased submucosal and epithelial expression of IL-17RA

**Fig 3:**
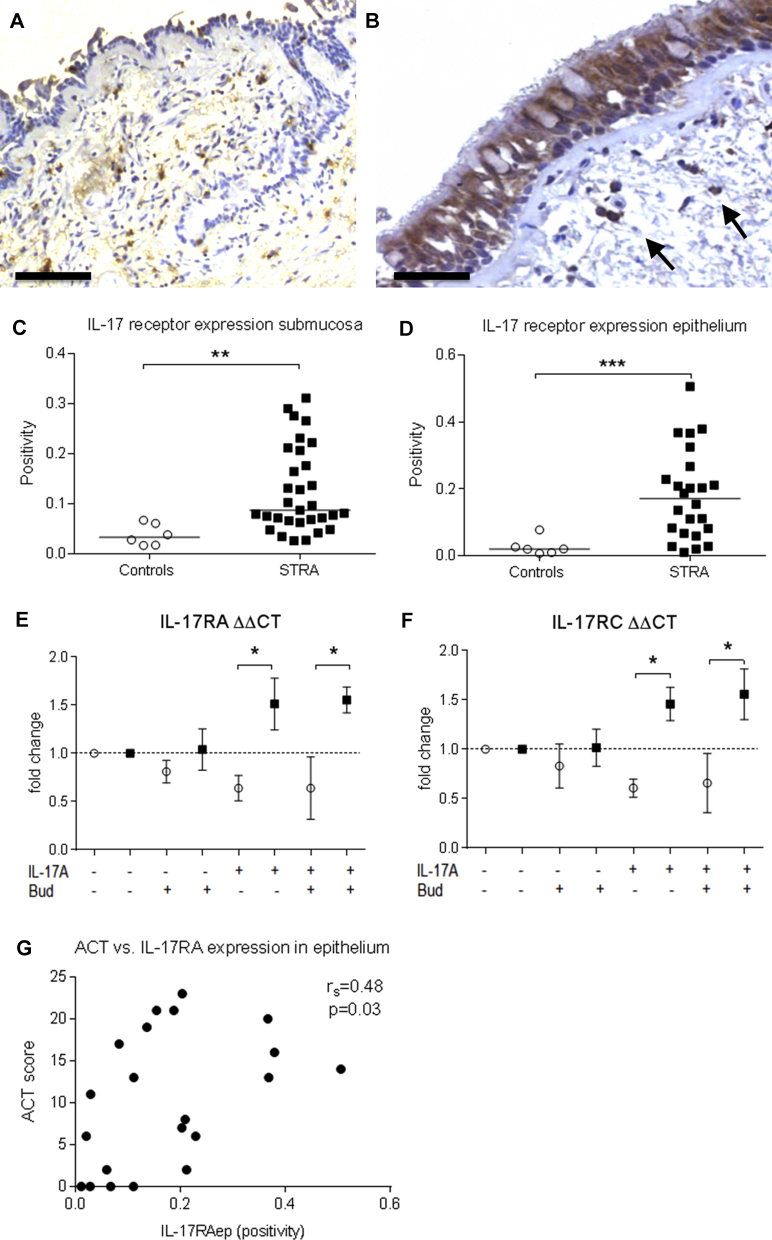
Representative micrographs of immunohistochemical stain for IL-17RA in controls without asthma and patients with STRA (**A** and **B,** respectively). Scare bar 100 μm (Fig 3, *A*) and 50 μm (Fig 3, *B*). Quantification of the expression (positive pixels per area) of IL-17RA in submucosa (controls without asthma n = 6, STRA n = 31) and epithelium (controls without asthma n = 6, STRA n = 24) (**C** and **D,** respectively). mRNA expression of IL-17RA and C in PBECs after stimulation with IL-17A and budesonide (Bud) (controls without asthma n = 3, STRA n = 3) (**E** and **F,** respectively). Correlations between ACT score and IL-17RA expression in epithelium **(G)** (n = 21). Statistical significance between controls without asthma and children with STRA was tested using Mann-Whitney test and correlation analysis was performed using Spearman rank test, where a *P* value of less than .05 was considered significant. *P < .05, **P < .01, and ***P < .001.

We further wanted to investigate the tissue expression of the receptor for IL-17A. IL-17RA was expressed in small mononuclear and polymorphonuclear cells in the submucosa in controls (Fig 3, *A*) and patients with STRA (Fig 3, *B*). Strong expression was also present in the epithelium of patients with STRA (Fig 3, *B*). There was significantly increased expression of IL-17RA in both submucosa (Fig 3, *C*) and epithelium (Fig 3, *D*) expressed as positive pixels per area (positivity) in patients with STRA compared with controls. Epithelial IL-17R expression remained elevated in patients with STRA even after excluding the patients with a positive bacterial culture or viral detection (*P* = .006). There was no difference in IL-17RA expression between the patients taking maintenance oral steroids and inhaled steroids compared with those only on inhaled steroids (*P* = .3).

Because intraepithelial IL-17R expression was significantly increased in patients with STRA, we determined how the bronchial epithelium responds to stimulation with IL-17A alone or in combination with budesonide. mRNA expression of IL-17RA and C was comparable between patients with STRA and controls at baseline, but expression was significantly increased in patients with STRA compared with controls following IL-17A stimulation alone, and with the addition of budesonide to cultures (Fig 3, *E* and *F*). Furthermore, better symptom control, measured using the asthma control test (ACT), correlated with increased IL-17RA expression in the epithelial compartment in children with STRA (Fig 3, *G*).

### IL-17A stimulation of bronchial epithelial cells from children with STRA induced IL-8 secretion

**Fig 4:**
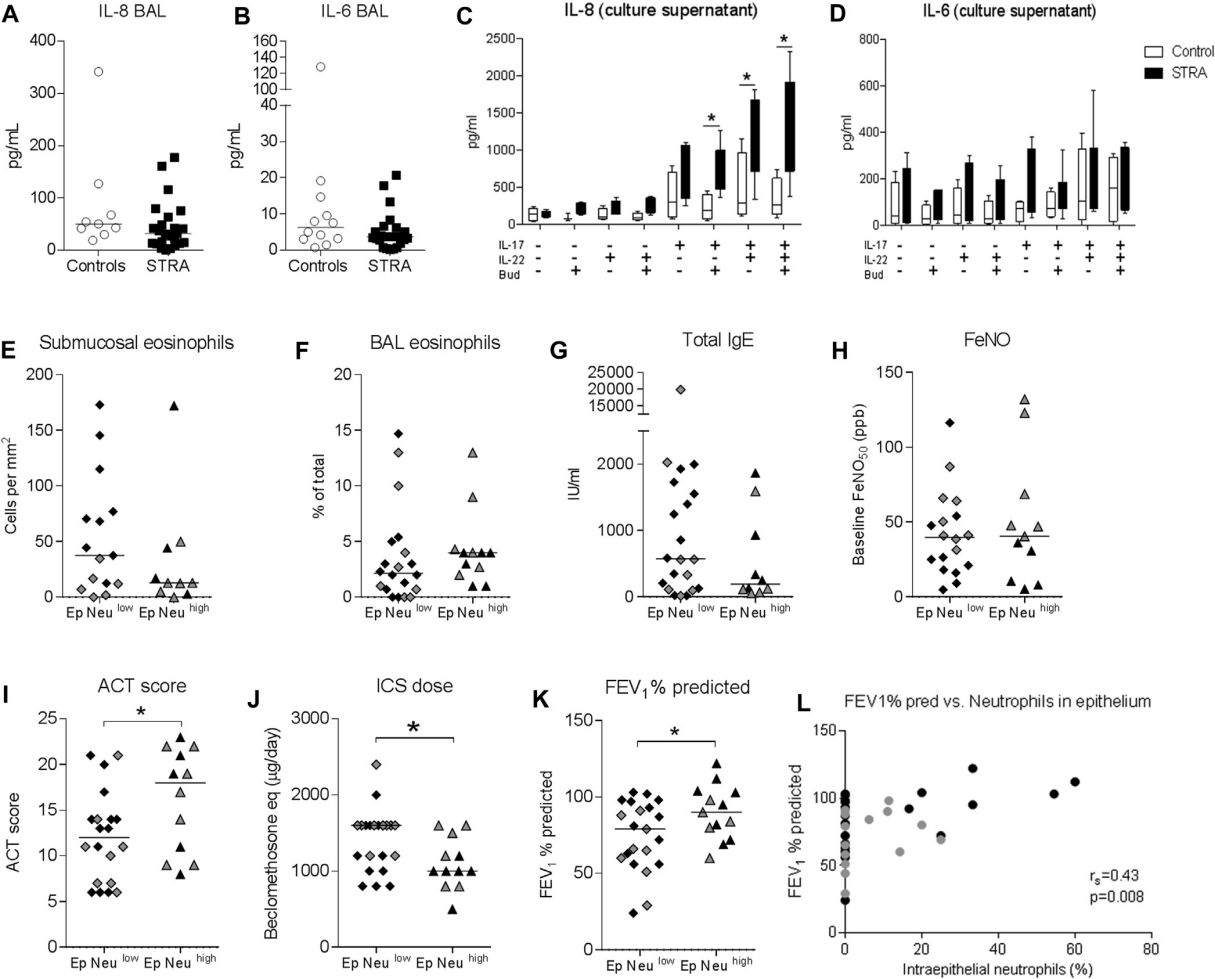
Levels of IL-8 **(A)** (controls without asthma n = 9, STRA n = 24) and IL-6 **(B)** (controls without asthma n = 12, STRA n = 23) in BAL. Levels of IL-8 **(C)** and IL-6 **(D)** in culture supernatant from PBECs after stimulation with IL-17A, IL-22, and budesonide (controls n = 4, STRA n = 7). Differences in submucosal eosinophils **(E)** (Neu^hi^ n = 10, Neu^lo^ n = 15), BAL eosinophils **(F)** (Neu^hi^ n = 12, Neu^lo^ n = 20), total IgE **(G)** (Neu^hi^ n = 10, Neu^lo^ n = 21), Feno**(H)** (Neu^hi^ n = 11, Neu^lo^ n = 18), ACT score **(I)** (Neu^hi^ n = 12, Neu^lo^ n = 20), dose of inhaled corticosteroids **(J)** (Neu^hi^ n = 12, Neu^lo^ n = 21), and FEV_1_ % predicted **(K)** within the group of children with STRA based on the presence of intraepithelial neutrophils (Neu^hi^ n = 13, Neu^lo^ n = 21). Correlations between FEV_1_ % predicted and proportion of intraepithelial neutrophils **(L)** (n = 36). Patients from the cohort previously published in Bossley et al^5^ are shown in gray. Statistical significance between controls and patients with STRA was tested using Mann-Whitney test and correlation analysis was performed using Spearman rank test, where a *P* value of less than .05 was considered significant. *F**eno*, Fraction of exhaled nitric oxide. *P < .05.

**Fig E1:**
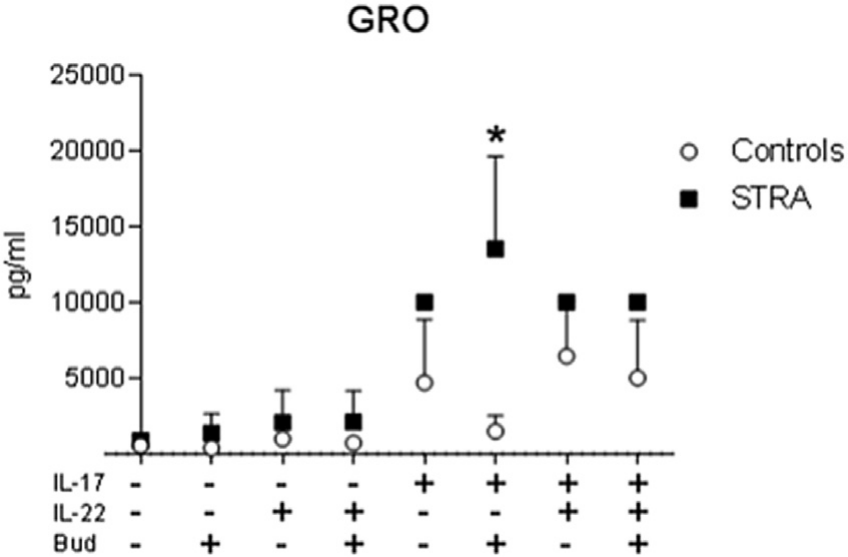
Measurement of GRO in culture supernatants from PBECs after stimulation with IL-17A, IL-22, and budesonide (Bud) (Fig E1, *A*) (controls without asthma n = 4, STRA n = 4). Statistical significance between controls without asthma and STRA was tested using Mann-Whitney test, where a *P value of less than .05 was considered significant.

Because IL-8 is a neutrophil chemoattractant and we had seen increased intraepithelial neutrophils in patients with STRA, levels of IL-8 were measured in BAL fluid. IL-6 was measured as a comparative marker of a general inflammatory response. BAL IL-8 and IL-6 levels were similar in patients with STRA and controls (Fig 4, *A* and *B*). This was unchanged after patients with a positive BAL bacterial culture or viral detection were excluded. IL-17A has previously been shown to induce IL-8 from bronchial epithelial cells.^28^ To investigate the relationship between IL-17A, epithelial cells, and neutrophils, we measured IL-8 secretion from PBEC culture supernatants following IL-17A and IL-22 stimulation, with or without budesonide. PBECs from patients with STRA secreted significantly higher amounts of IL-8 compared with cells from control patients. This effect was observed following stimulation with IL-17A alone, in combination with IL-22, or with IL-22 alone (Fig 4, *C*). Importantly, IL-8 secretion was unaffected by the presence of budesonide (Fig 4, *C*). In contrast, levels of epithelial IL-6 secretion were comparable between patients with STRA and controls (Fig 4, *D*). Levels of IL-13 (T_H_2 cytokine) and IFN-γ (T_H_1 cytokine) in epithelial cell culture supernatants were similar between patients with STRA and controls. However, levels of the neutrophil chemoattractant GRO were increased in patients with STRA compared with controls on IL-17A stimulation (see Fig E1 in this article's Online Repository at www.jacionline.org).

**Fig E3:**
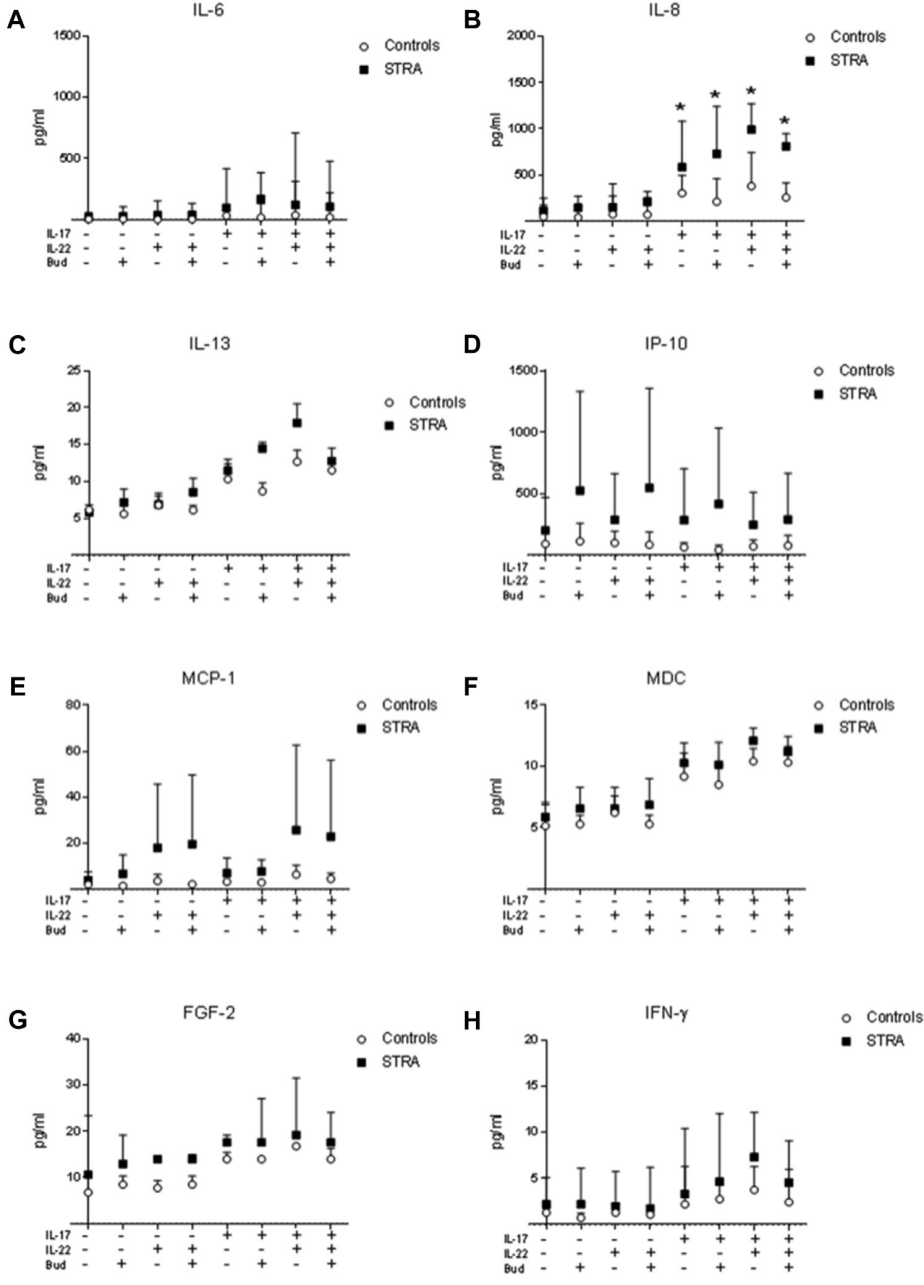
Measurement of IL-6 **(A),** IL-8 **(B),** IL-13 **(C),** IP-10 **(D),** MCP-1 **(E),** MDC **(F),** FGF-2 **(G),** and IFN-γ **(H)** in culture supernatants from PBECs after stimulation with IL-17A, IL-22, and budesonide (Bud) (controls without asthma n = 4, STRA n = 4) using Milliplex assay. Statistical significance between controls without asthma and STRA was tested using Mann-Whitney test, where a *P value of less than .05 was considered significant.

To further investigate the link between increased IL-17A receptor expression, intraepithelial neutrophils, and a clinical phenotype, correlations with clinical parameters were performed. We found no correlations with any confounding factor such as age or body mass index in children with STRA. Neither was there any association between intraepithelial neutrophils and parental smoking status or BAL bacteriology/virology (Table II). Tissue expression of IL-17A and IL-17RA was also compared in patients with and without intraepithelial neutrophils. Although no significant differences were found, there was a trend for increased epithelial IL-17RA expression in the group with intraepithelial neutrophils (see Fig E3 in this article's Online Repository at www.jacionline.org). Two subpopulations of children with STRA were apparent on the basis of the presence or absence of intraepithelial neutrophils. Patients were therefore divided into intraepithelial Neutrophil^high^ and Neutrophil^low^ groups. There were no differences in the number of submucosal, epithelial, or BAL eosinophils (Fig 4, *E* and *F*), total IgE (Fig 4, *G*), or fraction of exhaled nitric oxide (Fig 4, *H*) between the Neutrophil^high^ and Neutrophil^low^ groups. There was no relationship between reticular basement membrane thickness, a marker of airway remodeling, and intraepithelial neutrophils (see Fig E5, *E*, in this article's Online Repository at www.jacionline.org). However, the Neutrophil^high^ patients with STRA had a significantly higher ACT score (Fig 4, *I*) and were prescribed lower dose maintenance inhaled corticosteroids compared with the Neutrophil^low^ patients with STRA. FEV_1_ %predicted (Fig 4, *K*) was significantly higher in the Neutrophil^high^ patients with STRA, and the proportion of intraepithelial neutrophils positively correlated with FEV_1_ %predicted (Fig 4, *L*).

**Table II:** Neutrophil, IL-17A, and IL-17RA quantification in relation to BAL bacteriology, virology, and parental smoking status

Parameter	STRA (n = 51)	Controls without asthma (n = 15)	*P* value
BAL bacteriology (positive)	8 of 51 (15.4%)	1 of 15 (6.6%)	
BAL virology (positive)	5 of 51 (9.8%)	1 of 15 (6.6%)	
Parental smoking (positive)	15 of 41 (37%)		
Cotinine levels (ng/mL)∗	1.3 (1-4.8)		
IL-17A (submucosa)†	0.09 (0.03-0.3)	0.03 (0.02-0.07)	.001
IL-17RA (submucosa)‡	20 (0-291)	9 (0-47)	.4
IL-17RA (epithelium)‡	0.2 (0.01-0.5)	0.02 (0.007-0.08)	.0006
Neutrophils (submucosa)†	8 (0-202)	8 (0-36)	.9
Neutrophils (epithelium), %	0 (0-60)	0 (0-0)	.04
Eosinophils (submucosa)	54 (4-263)	8 (0-56)	.01
Eosinophils (epithelium), %	0 (0-0)	0 (0-0)	>.999

Comparison of intraepithelial neutrophils in STRA groups based on:			
	Positive	Negative	
BAL bacteriology	0 (0-17)	0 (0-60)	.7
BAL virology	8 (0-17)	0 (0-60)	.3
Parental smoking	0 (0-20)	0 (0-60)	.9

Data presented as median (range).

∗ >4.8 ng/mL indicates exposure to tobacco smoke and >50 ng/mL is indicative of active tobacco smoking.

† Cells per mm^2^.

‡ Positivity (positive pixels per all pixels). Differences between groups were assessed by Mann-Whitney test where *P* < .05 is significant.

## Discussion

We have shown that a subgroup of children with STRA have increased intraepithelial neutrophils compared with younger controls without asthma, which, surprisingly and contrary to our hypothesis, was associated with better FEV_1_ %predicted, symptom score, and lower maintenance inhaled steroids. In contrast, there were no intraepithelial eosinophils apparent in patients with STRA. Although there was no difference in tissue or luminal IL-17A, pediatric patients with STRA exhibited increased submucosal and epithelial expression of IL-17R. Collectively, our data indicate a potential beneficial role for intraepithelial neutrophils in this subphenotype of asthma.

The association between intraepithelial neutrophils and higher FEV_1_, higher ACT score, and less inhaled corticosteroid treatment suggests that neutrophils specifically within the epithelium may have a protective role in this asthma phenotype. Most previous studies were in adult patients with asthma and very few (including ourselves) have looked at the specific localization of neutrophils within the airway.^6^, ^7^, ^29^ Having found a relationship between intraepithelial neutrophils and lung function in the children reported here, we retrospectively quantified epithelial neutrophils in archived biopsies from our previously published cohort.^5^ The biopsies from the older cohort had not previously been assessed for intraepithelial neutrophils and confirmed an association between intraepithelial neutrophils and better spirometry, a significant strength of the study. Unlike intraepithelial neutrophils, eosinophils were not present within the epithelium of children with STRA. However, the increased eosinophils in the submucosa and lumen that we have reported previously^5^ were confirmed here (Fig 1, *A* and *B*). Although we cannot confirm a direct functional role of neutrophils in pediatric STRA, the localization of these cells within the epithelium may point to a specific role in host defense in this phenotype. Data from adults suggest that there may be at least 2 phenotypes associated with airway neutrophilia: beneficial as a response to infections, or harmful potentially driven by environmental irritants such as tobacco smoke.^30^ Exposure to viruses, bacterial endotoxins, and air pollution are common triggers of a neutrophil-rich inflammation and consequently could lead to asthma symptoms. Other studies have shown increased numbers of intraepithelial neutrophils in various bacterial, viral, and fungal infections as well as in cystic fibrosis and chronic obstructive pulmonary disease.^31^, ^32^, ^33^, ^34^, ^35^ However, we found no association between intraepithelial neutrophils and BAL bacteriology/virology or parental smoking status. Only 1% of bacteria can be cultured^36^; therefore, we cannot rule out that the children had other infections or an altered microbiota that we could not detect.^37^ It is also important to highlight that the children in this study were clinically stable and had at least 2 weeks since any exacerbation at the time of bronchoscopy. Because neutrophils have a high turnover and short lifespan, it is likely that the neutrophils observed within the epithelium in these patients are inherently present as opposed to recruited in response to an insult during an exacerbation.

There is an increasing need to find biomarkers that will indicate subgroups of patients (T_H_2-low, Neutrophil^high^) to enable effective personalized therapies. Other studies have identified that BAL and sputum neutrophils are more abundant in patients with severe asthma.^7^, ^38^ In a study investigating the molecular phenotype of severe asthma in children, both neutrophils and eosinophils were found to be elevated in BAL fluid from children with severe asthma compared with adult controls.^39^ These children also had high levels of GRO and IL-8 in BAL fluid.^39^ However, the specific location of neutrophils in the subcompartments of the bronchial wall was not investigated. The neutrophils in our study are intraepithelial and hence unlikely to result in a predictive biomarker in the periphery or airway lumen. However, molecules that represent intraepithelial neutrophils could be investigated in induced sputum in future studies. Unfortunately, we were unable to collect sputum from enough patients with intraepithelial neutrophils to undertake such an analysis.

It is possible that the intraepithelial neutrophils might be migrating through the epithelium into the lumen.^33^, ^34^, ^35^ But the increased epithelial expression of IL-17R, as well as the increased local epithelial production of neutrophil chemoattractants including IL-8 and GRO that we have demonstrated, suggest that neutrophils are trapped in the epithelial layer in children with STRA. Although speculative and further investigation is warranted, this might indicate an epithelial microenvironment that is dependent on altered expression of epithelial adhesion molecules that results in trapping of neutrophils in the epithelial layer.^36^, ^37^

There is scant information regarding the role of IL-17A in pediatric STRA. Higher numbers of IL-17A–positive cells have been found in the bronchial submucosa from adults with severe asthma compared with controls.^8^ IL-17 mRNA and numbers of IL-17–positive lymphocytes are also increased in BAL from patients with asthma.^10^, ^11^, ^40^ A recent study that has investigated the T_H_17 high phenotype in adult asthma has shown that a T_H_17 high signature is associated with steroid-dependent moderate-to-severe asthma and eosinophilia.^41^ But despite this signature there were no significant differences in lung function between patients. In addition, when IL-17 was blocked in a murine model of house dust mite–induced allergic airways disease, there was no impact on lung function, eosinophils, or neutrophils.^41^ Other studies have shown that T_H_17-mediated airway inflammation is steroid resistant.^42^ Furthermore, Nanzer et al^43^ reported that PBMCs from adults with severe asthma exhibited increased levels of T_H_17 cytokines, which were not inhibited by steroids.

We were careful to ensure all reagents were IL-17A specific, eliminating effects of contamination with IL-17F. Although significantly higher serum levels of IL-17A have been reported in children with asthma exposed to diesel exhaust particles,^44^ we found no increase in IL-17A in BAL fluid. Importantly, IL-17A levels were detectable, but similar between patients with STRA and controls, suggesting little influence from dilution effects of BAL. However, this might be because samples were collected during stable disease rather than following challenge or during disease exacerbation. After performing a double stain with IL-17A and the major leukocyte populations, we found that the major cell types in the submucosa expressing IL-17A were CD3+ lymphocytes and neutrophils. However, neither the epithelial cells nor infiltrating leukocytes within the epithelium were IL-17A positive. In contrast, our patients with STRA did show enhanced IL-17RA immunoreactivity in submucosa and epithelium as well as increased mRNA expression of IL-17RA and C on stimulation of PBECs with IL-17A when compared with controls without asthma. IL-17A and IL-17F both signal through IL-17RA,^25^ and have fundamental roles as neutrophil chemoattractants.^26^, ^33^ However, we found no association between neutrophils or IL-17A levels and infection in our patients, albeit molecular microbiological techniques were not used.

Deficiency in IL-17RA results in impaired neutrophil responses to allergens in mouse models.^45^, ^46^ Elevation of IL-17RA and C suggests a role in neutrophil migration, either via a direct response to IL-17A or via effector molecules such as IL-8. Although no significant difference was found between the patients with STRA with or without intraepithelial neutrophils with regard to IL-17A and IL-17RA expression in submucosa or epithelium, there was an indication of higher epithelial IL-17RA expression in the Neutrophil^high^ patients. We found no increased production of IL-6 in our study, which indicates a specific IL-17A–driven epithelial response, characterized by the neutrophil chemoattractants IL-8 and GRO, which were significantly increased with IL-17A stimulation of PBECs from patients with STRA compared with controls. These findings question the rationale for treating children with STRA with anti–IL-17A antibody. An mAb against IL-17RA, Brodalumab, has shown no benefit in adult patients with moderate-to-severe asthma^47^ and our data suggest that its use in children with STRA is unlikely to be beneficial.

All the patients with STRA in our study were symptomatic and had poor control despite a high dose of inhaled steroids. Corticosteroids have been shown to increase airway neutrophils in asthma^48^, ^49^ and molecules that are associated with suppression of neutrophil apoptosis are upregulated by glucocorticosteroids. This may be an unwanted effect of asthma therapy. In contrast, our data indicate that patients with lower epithelial neutrophils were prescribed higher doses of maintenance inhaled steroids, suggesting an altered relationship between intraepithelial neutrophils and glucocorticosteroids. This suggests that the neutrophil low group may be less sensitive to steroids because group members were on a higher median dose as a group.

**Fig E4:**
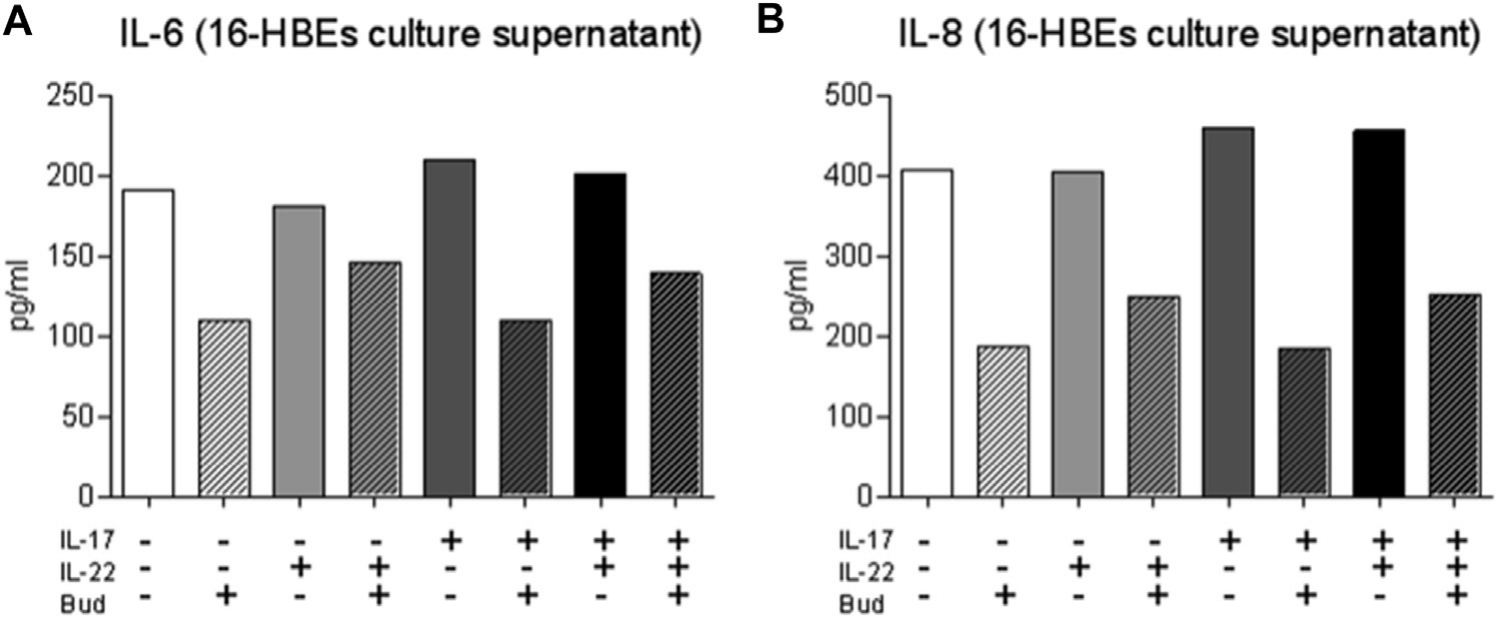
Measurement of IL-6 **(A)** and IL-8 **(B)** in culture supernatants from 16HBEs after stimulation with IL-17A, IL-22, and budesonide (Bud) using ELISA.

**Fig E5:**
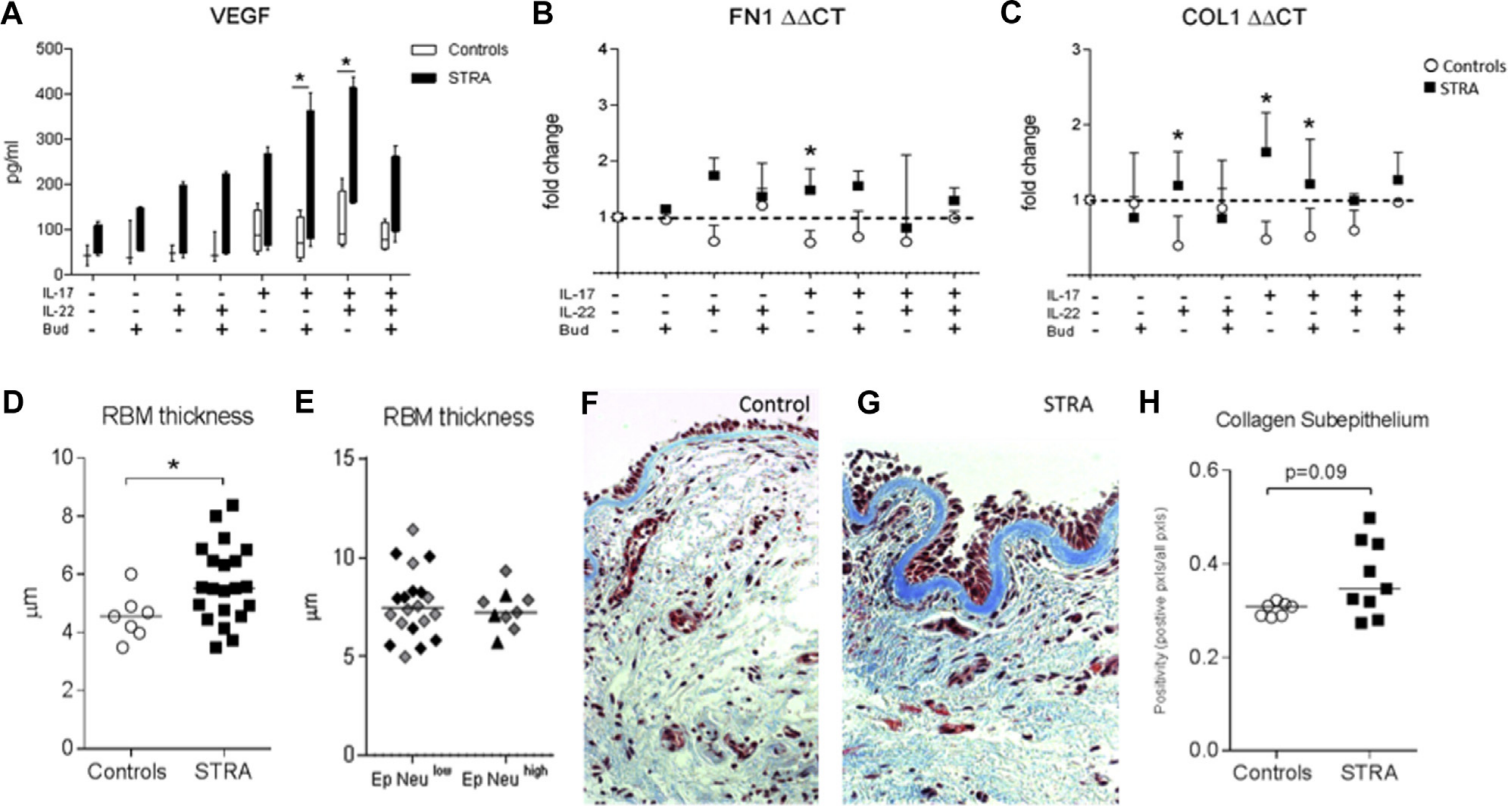
The effect of IL-17A on airway remodeling parameters. Measurement of VEGF in culture supernatants from PBECs after stimulation with IL-17A, IL-22, and budesonide **(A)** (controls without asthma n = 4, STRA n = 4). mRNA expression in PBECs after stimulation with IL-17A, IL-22, and budesonide of fibronectin **(B)** and collagen I **(C)** (controls without asthma n = 3, STRA n = 3). RBM thickness in controls without asthma compared with STRA **(D)** (controls without asthma n = 7, STRA n = 21) and RBM thickness within the STRA group when divided into Neutrophil^high^ subgroup vs Neutrophil^low^ subgroup **(E)**. Representative micrographs of bronchial biopsies stained with Masson's trichrome staining for collagen (blue) (**F** control and **G** STRA). Subepithelial collagen deposition **(H)** (nonasthmatic controls n = 7, STRA n = 9) in bronchial biopsies from controls without asthma and children with STRA expressed as positive pixels per area. Statistical significance between controls without asthma and STRA was tested using Mann-Whitney test, where a *P value of less than .05 was considered significant.

We have shown an association between increased IL-17RA expression and STRA. IL-17A–induced epithelial secretion of IL-8 was also unaffected by the presence of budesonide. Previous studies have also shown a lack of effect of dexamethasone on IL-17A production from PBMCs in both mice and humans.^27^, ^43^ These data suggest a lack of association between IL-17A and steroids in patients with severe asthma. Interestingly, it has been shown that the release of GRO and IL-8 induced by IL-17 in the bronchial epithelial cell line 16-human bronchial epithelium is sensitive to hydrocortisone treatment.^50^ We have found similar results with this immortalized cell line (see Fig E4 in this article's Online Repository at www.jacionline.org), confirming critical functional differences between PBECs and 16-human bronchial epitheliums and emphasizing the importance of using primary cells from patients with asthma.

The strengths of this study include the large number of carefully characterized children with STRA, a phenotype of asthma that has been little studied, and the comparison of clinical parameters with morphological studies and functional *in vitro* assays using primary airway epithelial cells from the same patients. However, we acknowledge some limitations. We did not include a control group of mild to moderate asthma, nor did we include true healthy controls. Although an invasive procedure involving a general anesthetic cannot be ethically justified for research studies in children, the controls included did not have lower respiratory tract symptoms, and when we have used similar patients previously we have found meaningful group differences.^5^, ^51^, ^52^ In addition, the controls were younger because isolated upper airway problems are less common in school-aged children.

In conclusion, our study shows that children with STRA compared with younger controls without asthma have an exaggerated epithelial response to IL-17A, with increased expression of IL-17RA in the airway submucosa and epithelium. PBECs from children with STRA responded to IL-17A stimulation with elevated production of the neutrophil-attracting mediators IL-8 and GRO compared with controls. Furthermore, increased numbers of neutrophils were found only in the epithelial compartment in a subgroup of children with STRA and this finding was associated with better lung function, better symptom control, and lower dose maintenance inhaled steroids. The critical role of the bronchial epithelium and its interactions with airway leukocytes in determining downstream functional effects has been highlighted. Our study shows that there are 2 subgroups within the pediatric STRA phenotype: intraepithelial Neutrophil^high^ and Neutrophil^low^, further demonstrating that STRA is heterogeneous and requires careful subphenotyping to identify optimal personalized molecular therapies.

**Clinical implications**

Our findings suggest that airway neutrophilia in pediatric patients with STRA may be a beneficial host response, and thus should be enhanced not reduced therapeutically.

## Figures and Tables

**Fig E2:**
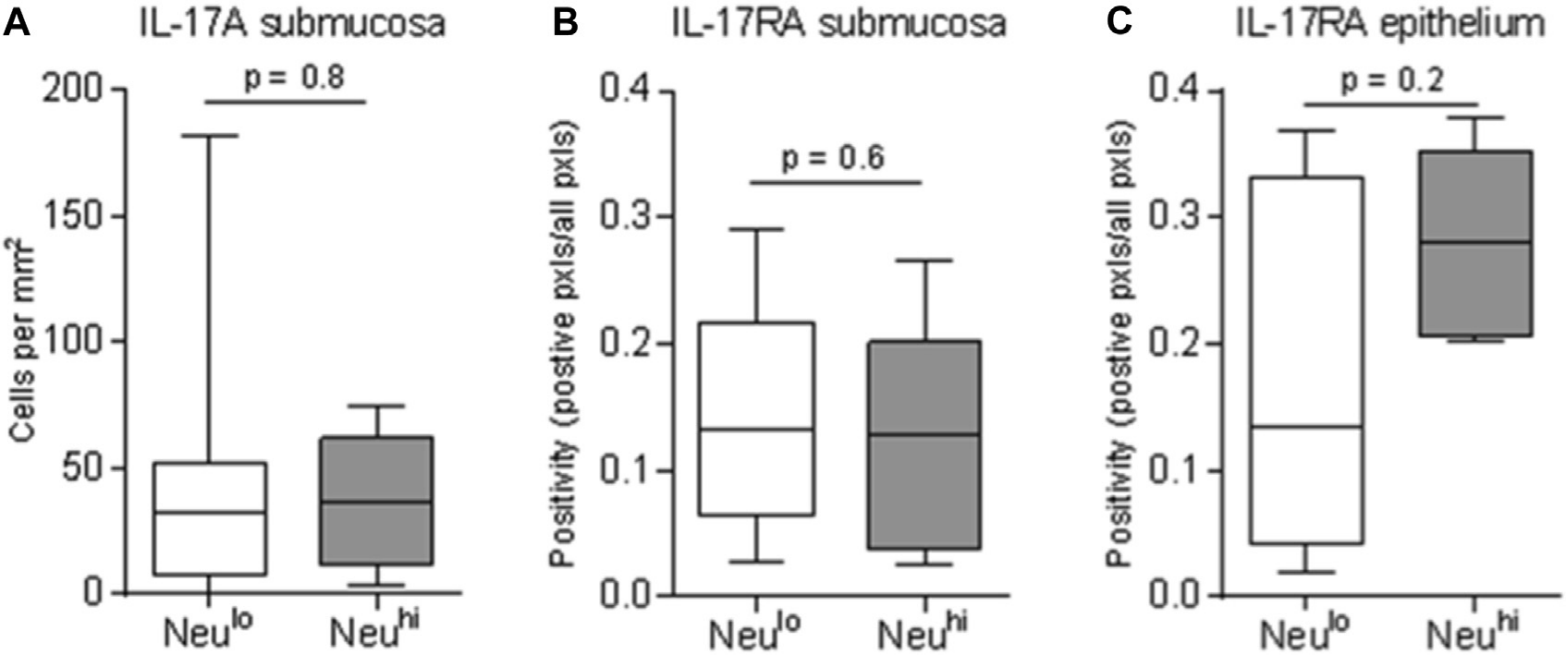
Tissue density of IL-17A in submucosa **(A),** IL-17RA in submucosa **(B),** and IL-17RA in epithelium **(C)** in bronchial biopsies from children with STRA divided into Neutrophil^high^ vs Neutrophil^low^ groups. Statistical significance was tested using Mann-Whitney test, where a *P* value of less than .05 was considered significant. *Neu*, Neutrophil.

**Fig E6:**
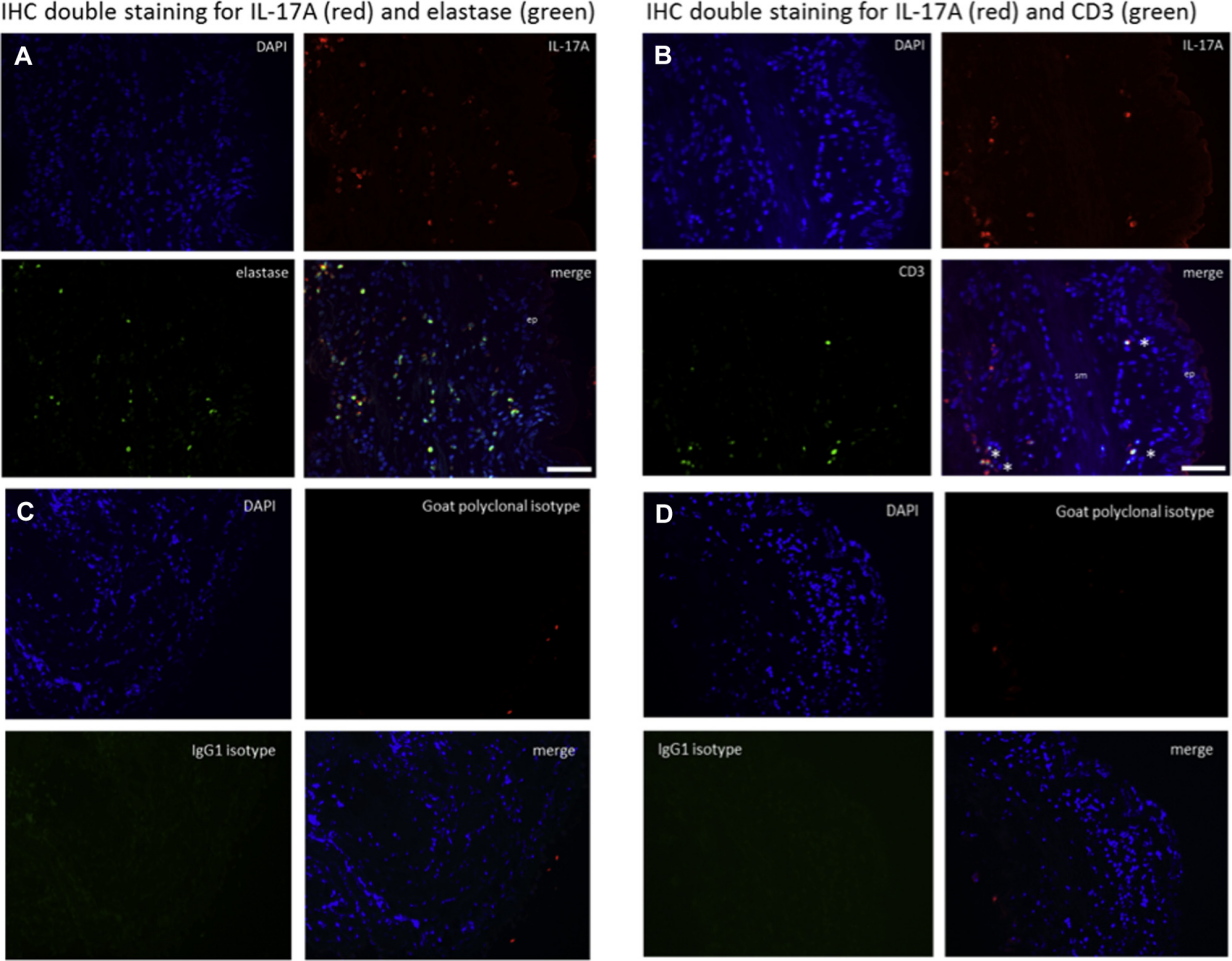
Immunohistochemical double staining of bronchial biopsies of IL-17A (red, AlexaF 555) and elastase **(A)** or CD3 **(B)** (green, AlexaF 488). Corresponding isotype controls are shown in **C** and **D.** Nuclei are stained blue with DAPI. *DAPI*, 4'-6-Diamidino-2-phenylindole, dihydrochloride; *IHC*, immunohistochemistry. Scale bar: 100 μm.

**Table E1:** Demographic characteristics of patients with STRA and controls undergoing bronchoscopy in validation cohort collected between 2007 and 2009

Characteristic	STRA (n = 21)	Healthy control (n = 5)	*P* value
Atopy	21 of 21 (100%)	0 of 5 (0%)	
Male:female	14:7	3:2	
Age (y)	11.2 (8 to 16)	11.2 (9 to 16)	.80
Weight (kg)	42.4 (24.1 to 74.0)	44.7 (37.8 to 60.2)	.96
Height (cm)	141 (114 to 159)	160 (144 to 168)	.054
Total IgE (IU/mL)	415 (16 to 18645)	—	
BAL neutrophils (%)∗	3 (0 to 19)	1.3 (0 to 14)	.22
BAL eosinophils (%)†	3 (0 to 51)	0 (0 to 10)	.33
ACT score‡	11 (6 to 22)	—	
Baseline % predicted FEV_1_§	69 (29 to 98)	98 (78 to 106)	.0072
Number FEV_1_ “normal” (>80% predicted)	6 of 21 (28.6%)	4 of 5 (80%)	
Baseline % predicted FVC	99.5 (63 to 133)	93 (89 to 116)	.56
Baseline bronchodilator reversibility (%)	13.3 (−2 to 135)	—	
Medications			
Daily dose inhaled‖ corticosteroid (μg/d) budesonide equivalent	1600 (800 to 3200)	0	
Systemic corticosteroids	12 of 21 (57%)	0	
Daily dose (mg/d)	6.88 (5 to 40)	0	

Data presented as median (range).

∗ BAL neutrophils (<3% is normal).

† BAL eosinophils (<3% is normal).

‡ Asthma control test (>19 is normal).

§ FEV_1_: presented as percentage predicted.

‖ ICS: inhaled corticosteroids/d. Differences between groups were assessed by Mann-Whitney test where *P* < .05 is significant.

**Table E2:** Clinical diagnosis and reason for bronchoscopy of controls without asthma

Clinical diagnosis	Controls without asthma (n = 15)
Elective cardiac catheter	2
Unexplained hypoxia	1
Reflux	2
Tracheal stenosis	2
Chronic dry cough	5
Hemoptysis	2
Laryngotracheomalacia	1

Data presented as number of patients.

## Methods

### Subjects

Fifty-one school-aged children with STRA who were undergoing a clinically indicated bronchoscopy were recruited from the Royal Brompton Hospital between 2009 and 2015. Patients remained poorly controlled despite being prescribed high doses of inhaled steroids (800 μg/d budesonide or equivalent) and receiving additional long-acting β-agonists, and current or a previous failed trial of montelukast (Table I). Twelve percent of the patients with severe asthma were also taking regular oral steroids. Only children with true STRA were included in the study.E1, E2, E3 Those with difficult asthma, in whom underlying modifiable factors such as persistent allergen exposure, poor adherence, and/or inhaler technique that might contribute to poor asthma control, were excluded.^E4^ The remaining children with STRA underwent invasive analysis with bronchoscopy, bronchial brushings, BAL, and endobronchial biopsies to characterize airway pathology and develop customized treatment plans. *Atopy* was defined as 1 or more positive specific IgE RAST (≥0.34 kU/L) or 1 or more positive skin prick test results to aeroallergens. Asthma control was assessed by using the ACTE5, E6; *poor control* was defined as a score of less than 20 of 25. Spirometry and bronchodilator reversibility defined as a greater than 12% change from baseline FEV_1_ were measured according to American Thoracic Society/European Thoracic Society guidelines.E7, E8 Exhaled nitric oxide measurements at a flow rate of 50 mL/s were made with a chemiluminescence analyzer (NIOX; Aerocrine AB, Solna, Sweden) in accordance with American Thoracic Society/European Thoracic Society guidelines.^E9^ Clinical details of children with STRA are presented in Table I. Fifteen control subjects without asthma were either having a bronchoscopy to investigate upper airway symptoms and agreed to extra research samples being taken or were undergoing general anesthesia for cardiac symptoms and agreed to have a research bronchoscopy at the same time (see Table I and Table E2 for details). The study was approved by the Brompton and Harefield National Heart and Lung Institute's Ethics Committee, and informed parental consent and child assent were obtained.

Findings relating to epithelial inflammation in this group were confirmed in biopsies already obtained from a previously published cohort of children with STRA (n = 21) and controls without asthma (n = 5) (Table E1). The inclusion criteria in this cohort were the same as for the patients described above (see also Bossley et al^E1^). Children aged 5 to 16 years with STRA were included. Definitions of uncontrolled disease and entry criteria were as follows:

1. Persistent (most days for ≥3 months) chronic symptoms (use of short-acting β2-agonists ≥3 times per week) of airway obstruction despite high doses of inhaled corticosteroids (≥800 μg/d budesonide equivalent) and/or regular oral corticosteroids, long-acting β2-agonists, and current (or previous failed trial of) montelukast.
2. Recurrent severe exacerbations requiring 1 or more admission to the intensive care unit or 2 or more hospital admissions requiring intravenous medications or 2 or more courses of oral corticosteroids in the past year despite therapy for persistent symptoms as described above.
3. At least 1 very sudden (≤6 hours) severe attack (requiring hospitalization) without warning despite therapy for persistent symptoms, as described above.

Evaluations were performed to assess medication, dose, device and technique, atopic status, asthma understanding, adherence, environment, and any psychosocial issues. After assessment, those classified as true STRA were further investigated. The study was approved by the local research ethics committee, and all procedures were performed after obtaining written informed parental consent and, where appropriate, child assent.

### Epithelial cell culture and stimulation

Epithelial brushings were obtained at bronchoscopy using a fiberoptic bronchoscope (Olympus, Keymed, United Kingdom). A sterile single-sheathed nylon cytology brush was used to sample epithelial cells from the bronchial mucosa of the second- and third-generation bronchi. Cells were transferred to 5-mL bronchial epithelial growth medium (BEGM, Lonza, Walkerville, Md) and the samples were centrifuged at 1500*g* for 5 minutes to pellet the cells. Primary cultures were established by reconstituting the pellet in BEGM and cells were seeded into collagen-coated T25 tissue culture flasks containing 5 mL BEGM. Cultures were routinely tested for mycoplasma infection.

Primary pediatric bronchial epithelial cells were grown to 80% to 90% confluence in 12-well plates. On the day of stimulation, the growth medium was replaced with bronchial epithelial basal medium (Lonza) 4 hours before the start of the experiment. The primary epithelial cells were used at passage 3 for all experiments. PBECs were stimulated with recombinant human IL-17A (eBioscience) and IL-22 (eBioscience) at 10 ng/mL alone, or with added budesonide at a final concentration of 10^−8^ mol/L. All experiments were done in triplicate. After 24-hour cell culture, supernatants were taken, and cell lysates were prepared in RIPA buffer containing protease inhibitor tablets (Roche Diagnostics, Mannheim, Germany). Samples for mRNA extraction were undertaken in separate 12-well plates and cells were harvested in Trizol (Sigma, St Louis, Mo) after 8-hour stimulation.

Sixteen human bronchial epitheliums (HBEs), a differentiated SV-40–transformed bronchial epithelial cell line, was used in the same way as the primary human bronchial epithelial cells and stimulated as described above with IL-17, IL-22, IL-17, and IL-22 in combination with or without budesonide.

### Quantification of cytokines

Cell culture supernatant was collected and cytokines were analyzed using ELISA and Milliplex. Milliplex human cytokine panel I and II (HCYP2MAG-62K and MPXHCYTO-60K, Millipore Corporation, Billerica, Mass) were used to detect levels of IL-4, IL-5, IL-6, IL-8, IL-9, IL-10, IL-13, IL-17, IP-10, MCP-1, MDC, RANTES, TNF-α, VEGF, eotaxin, eotaxin-2, eotaxin-3, fibroblast growth factor 2 (FGF2), growth-regulated oncogene (GRO), thymus and activation regulated chemokine (TARC/CCL17), stem cell factor (SCF), thymic stromal lymphopoietin (TSLP), and IL-33 according to manufacturer's instructions. Levels of IL-6 and IL-8 were confirmed with standardized sandwich ELISAs, according to the manufacturer's protocol (88-8086 and 88-7066, eBiosciences).

### Cytomix

BAL fluid cytokine quantification was performed using Flowcytomix Human T_H_1/T_H_2/T_H_9/T_H_17/T_H_22 13 plex multiplex (eBioscience) to detect levels of IFN-γ, IL-1β, IL-2, IL-4, IL-5, IL-6, IL-9, IL-10, IL-12 (p70), IL-13, IL-17A, IL-22, and TNF-α according to manufacturer's instructions. Samples were assayed using the BD Fortessa flow cytometer. Data analysis was performed using Flowcytomix software (eBioscience) and GraphPad Prism (V.6 for Mac OS X, GraphPad Software Inc, San Diego, Calif).

### Fluorescence-activated cell sorting

Lymphocytes used for compensations and fluorescence minus ones were extracted from peripheral blood using a density gradient medium (LymphoprepTM). Cell concentration was optimized to 2 to 4 × 10^5^ cells per 100 μL. Cells from the airway lumen were obtained after centrifuge of BAL fluid to obtain a cell pellet. Cells were resuspended in RPMI complete and plated at 2 to 4 × 10^5^ cells per 100 μL. Cells were stimulated for 4 hours with phorbol 12-myristate 13-acetate (PMA), ionomycin, and Brefeldin A. Cells were stained for extracellular markers CD3 (1:50), CD4 (1:50), CD8, CD161, and γδTCR and intracellular staining for IL-17A (eBiosciences, mouse antihuman 5 μL per well).

Samples were assayed using the BDFortessa flow cytometer (BD, Oxford, United Kingdom). Data were analyzed using FlowJo (V.9.2, TreeStar Inc, Ashland, Ore) and GraphPad Prism (V.6 for Mac OS X, GraphPad Software Inc).

### RNA extraction and real-time PCR

Total RNA was extracted from epithelial cells by using the Qiagen RNeasy Mini Kit (Qiagen, Hilden, Germany). cDNA was synthesized from 500 ng of total RNA and analyzed by using High Capacity cDNA Reverse Transcription Kit (Applied Biosystems). Reactions were run with TaqMan primers against IL-17RA, IL-17RC, Fibronectin, Collagen I (Applied Biosystems), and TaqMan Fast Advanced Master Mix (Applied Biosystems) according to manufacturer's directions on a Viaa-7 (Life Technologies, Basingstoke, United Kingdom) instrument and gene expression was analyzed using the change-in-threshold ΔΔCt- method.

### Histopathology

Endobronchial biopsies were processed to paraffin. Five-micrometer sections were stained with hematoxylin and eosin and used to assess morphology, and consecutive sections were used for Masson's trichrome staining (Sigma Aldrich) and immunohistochemical staining for IL-17A, IL-17RA, and neutrophil elastase. Human polyclonal goat anti–IL-17 antibody (R&D, Rocky Hill, NJ; dilution 1:50) and human monoclonal mouse anti–IL-17RA antibody (R&D; dilution 1:50) were detected by using Dako EnVision (K5007) or anti-goat horseradish peroxidase (Dako, Glostrup, Denmark). Staining was visualized with 3′,3′-diaminobenzidine (DAB kit, Vector Laboratories, Peterborough, United Kingdom) and counterstained with hematoxylin. Human monoclonal mouse antielastase (Dako, dilution 1:100) was used for detection of neutrophils. Primary was detected with Vector Laboratories ABC kit (AK5000) according to manufacturer's instructions and was visualized with Fast Red and counterstained with hematoxylin. IL-17A–positive cells were quantified in the submucosa and expressed as cells per square millimeter of tissue using ImageScope (Aperio, Vista, Calif). IL-17RA was quantified using ImageScope positive pixel count algorithm in the submucosa and epithelium and expressed as positive pixels divided by total number of pixels (positivity). Neutrophil density was quantified in the epithelium and the subepithelial compartment using ImageScope. Neutrophils in the submucosa were expressed as cells per square millimeter tissue and intraepithelial neutrophils as the proportion of intraepithelial neutrophils of all neutrophils present in the biopsy as well as number of cells per micrometer reticular basal membrane length. Because only a proportion of patients with STRA had intraepithelial neutrophils, we included biopsies from another cohort with similar patients (patients' details have been previously published^E1^) to be able to do a statistically assured comparison and correlation to FEV_1_ % predicted.

Double staining with immunofluorescence was used to simultaneously visualize IL-17A together with the following molecules: CD68 (1:200, Dako), elastase (1:200, Dako), tryptase (Dako), and CD3 (1:50, Dako). The markers were selected to represent multiple leukocyte populations that might express IL-17A. After antigen retrieval (citrate buffer, pH 6), sections were stained for IL-17A using specific and validated primary antibody (IL-17A, R&D Systems) and visualized by an Alexa-Flour 555–conjugated secondary antibody (Molecular Probes, Eugene, Ore). Next, antibodies against CD68, elastase, tryptase, or CD3 were added and visualized using an AlexaF-488–conjugated secondary antibody. Sections were mounted with Prolong gold mounting medium with 4′-6-diamidino-2-phenylindole, dihydrochloride (Molecular Probes) for visualization of cell nuclei.

### Statistical analysis

Nonparametric Mann-Whitney *U* tests and Kruskal Wallis test with Bonferroni *post hoc* test were used to detect differences between 2 groups or more than 2 groups, respectively, by using GraphPad Prism 6 software (GraphPad Software, La Jolla, Calif). Correlations were assessed by using the Spearman rank correlation test. Results were considered significant at a *P* value of less than .05.

## Results

### Stimulation of bronchial epithelial cells with IL-17A–induced secretion of GRO in children with STRA

Because IL-17A is associated with increased numbers of neutrophils in adults with asthma, levels of GRO were measured in BAL fluid as well as in PBEC culture supernatants following IL-17A and IL-22 stimulation, with or without budesonide. Epithelial GRO secretion from bronchial epithelial cells was significantly increased in patients with STRA compared with controls without asthma with IL-17A stimulation in combination with budesonide (Fig E1, *A*). No effect was seen with IL-22 stimulation alone.

Detectable levels of IL-6, IL-8, IL-13, IP-10, MCP-1, MDC, FGF-2, and IFN-γ were also found in cell culture supernatants using Milliplex but no difference was found between children with STRA and controls without asthma expect for IL-8, which confirmed the ELISA data (Fig E2, *B*). The other mediators in the Milliplex kit were below detection level.

### Correlations between specific serum IgE levels and clinical parameters, neutrophil numbers, and IL-17A/RA expression

We did not find any correlation of submucosal or intraepithelial neutrophils, IL-17A, and IL-17RA expression to specific IgE (sIgE) levels to any specific allergen nor to the sum of inhaled allergen or to the total sum of sIgE to all allergens. However, positive correlations were found between sum of sIgE to inhaled allergens and reticular basement membrane (RBM) thickness (rs = 0.43; *P* = .05) and sum of sIgE to inhaled allergens and submucosal eosinophils (rs = 0.63; *P* = .03).

### 16-HBEs cell line appears to be sensitive to treatment with budesonide

In contrast to the primary HBECs, the 16HBE cell line did not respond with increased IL-6 and IL-8 production on stimulation with IL-17A, IL-22, or in combination. However, they did display sensitivity to budesonide and showed decreased production of IL-6 and IL-8 on treatment with budesonide (Fig E3, *A* and *B*).

### IL-17A stimulation induced epithelial expression of remodeling mediators

Increased RBM thickness and airway smooth muscle mass are distinct features of children with STRA.^E1^ Because IL-17A and IL-22 have been associated with airway remodeling,E10, E11, E12 we measured epithelial secretion of remodeling mediators in response to stimulation with these cytokines. VEGF was significantly increased in children with STRA compared with controls without asthma when IL-17A and IL-22 were used in combination (Fig E5, *A*; *P* = .04). Fibronectin mRNA was increased in children with STRA compared with controls without asthma when cells were stimulated with IL-17A alone (*P* = .02; Fig E5, *B*), while collagen I was increased in children with STRA compared with controls without asthma when cells were stimulated with IL-22 (*P* = .02), IL-17 (*P* = .005), or IL-17 with budesonide (*P* = .05) (Fig E5, *C*). We confirmed our previous findings (1) that children with STRA have increased RBM thickness (*P* = .03; Fig E5, *E*). However, no significant difference in submucosal collagen deposition was found in bronchial biopsies from controls without asthma and patients with STRA (*P* = .09; Fig E3, *F* and *G*).
